# Estimated breeding values and association mapping for persistency and total milk yield using natural cubic smoothing splines

**DOI:** 10.1186/1297-9686-41-48

**Published:** 2009-11-05

**Authors:** Klara L Verbyla, Arunas P Verbyla

**Affiliations:** 1 Victorian Department of Primary Industries, Bundoora, VIC, 3083, Australia; 2 School of Agriculture, Food and Wine, The University of Adelaide, Adelaide, SA 5005, Australia; 3 Mathematical and Information Sciences, CSIRO, Urrbrae, SA 5064, Australia

## Abstract

**Background:**

For dairy producers, a reliable description of lactation curves is a valuable tool for management and selection. From a breeding and production viewpoint, milk yield persistency and total milk yield are important traits. Understanding the genetic drivers for the phenotypic variation of both these traits could provide a means for improving these traits in commercial production.

**Methods:**

It has been shown that Natural Cubic Smoothing Splines (NCSS) can model the features of lactation curves with greater flexibility than the traditional parametric methods. NCSS were used to model the sire effect on the lactation curves of cows. The sire solutions for persistency and total milk yield were derived using NCSS and a whole-genome approach based on a hierarchical model was developed for a large association study using single nucleotide polymorphisms (SNP).

**Results:**

Estimated sire breeding values (EBV) for persistency and milk yield were calculated using NCSS. Persistency EBV were correlated with peak yield but not with total milk yield. Several SNP were found to be associated with both traits and these were used to identify candidate genes for further investigation.

**Conclusion:**

NCSS can be used to estimate EBV for lactation persistency and total milk yield, which in turn can be used in whole-genome association studies.

## Background

For dairy producers, the accurate description of lactation curves is a valuable tool for selection and management. Lactation curves provide a description of milk yield performance, which make it possible to predict total milk yield from a single or several test days early in lactation. Thus, producers can make early management decisions based on the predicted individual production. Different mathematical equations have been proposed to model lactation curves. Usually such curves are modelled using parametric models with fixed or random coefficients, for example random regression models, Wood's Lactation Curve (the commonly applied gamma equations), Wilmink's Curve and Legendre polynomials. Alternatively, mechanistic models which describe the lactation curves based on the biology of lactation have been used [1]. In 1999, White and colleagues [2] proposed and demonstrated that Natural Cubic Smoothing Splines (NCSS) can model the features of lactation curves with greater flexibility than the traditional parametric methods. This has been further supported by the work of Druet and colleagues [3]. In addition, NCSS are particularly useful in an animal breeding setting since they can be incorporated into linear mixed models.

A lactation curve describes many important features of lactation and some of these features, namely time to peak, total milk yield and rate of decline after the peak yield, were examined in this study. The rate of decline in milk production after peak yield is the typical definition of milk yield persistency. High persistency is characterized by a slow rate of decline after peak yield, while low persistency is characterized by a high rate of decline after peak yield. Persistency has been reported to have a significant economic impact [4]. Highly persistent cows or cows with a flat lactation curve are reported to be more profitable because of fewer health and reproductive problems with less energy imbalance. The links between health disorders, fertility and persistency have been investigated with varied results [5,6].

Total milk yield is a well-known economically important trait. However, selection for high total milk yield has been shown to have detrimental health effects [7]. If an animal has a low persistency, selection for high milk yield can cause significant metabolic stress. In 2004, Muir and colleagues [8] have reported that selection for increased persistency might increase total yields without increasing disease incidences or fertility problems. Subsequently, Togashi and Lin [9,10] have investigated different selection strategies to maximize milk yield without decreasing persistency.

Although the definition of persistency is now generally agreed upon, methods of estimation still vary. In 1996, Gengler [11] provided a review of many common definitions of persistency, which included ratios of an early test day or period to late-lactation test-day or period and measures formulated to be independent of total yield. Other reported measures are the difference between one set day for peak yield (or the estimated breeding value (EBV) at this day) early in lactation and a test day late in lactation (or EBV at this day), or the sum of the yield or EBV over this time period. Novel approaches for calculating persistency have been presented by Druet and colleagues [12] and Togashi and Lyn [13]. Cole and VanRaden [14] and Cole and Null [15] have shown that routine genetic evaluations are feasible for persistency. Some of these methods assume one set day for peak yield for all animals, which in reality is not the case. Using NCSS allows the exact estimation of a unique peak day and yield at peak for each animal.

Many QTL and association studies have been conducted for total milk yield and a few QTL studies have investigated persistency. Such studies usually involved either the use of single markers or a genome scan to establish association with a specific trait. Whole-genome approaches have been developed, for example genetic random variable elimination (GeneRaVE) [16,17] and whole-genome average interval mapping (WGAIM) [18]. Whole-genome methods allow for background genetic effects by incorporating all markers, and thus all the associations between marker and trait are estimated simultaneously.

The first objective of this paper was to demonstrate that NCSS could be used successfully to estimate sire breeding values for two important features of the lactation curve, persistency and total milk yield, for a specific set of sires in a large Australian study. The second objective was to conduct an association study for both persistency and total milk yield using the calculated EBV, genotype information in the form of 7541 single nucleotide polymorphisms (SNP) and a maternal grandsire pedigree. The overall aim was to use a whole-genome association study to establish marker-trait associations.

## Methods

### Materials

Genotypic information was available for 383 Holstein Friesian (HF) progeny-tested bulls, which were selected on the basis of either high or low estimated breeding values for the Australian selection index. The index's primary emphasis is on protein production. Data on all these bulls' daughters and their contemporaries were extracted from the Australian Dairy Herd Improvement Scheme (ADHIS) database. The data set consisted of Holstein Friesian cows that calved during the period 1983 to 2006 and were in the same herd year and season as the daughters of the 383 genotyped sires. Records were removed when calving date was missing or when the test date was outside the 5 to 305 d in milk (DIM) period. Only first lactations were included since it has been demonstrated that genetic correlations for persistency between consecutive parities are high [19] (> 0.85 reported between the first two parities) despite previous results disagreeing with this study (see [19] for discussion of results). This data set contained over 15 millions test day records from the daughters of 38,381 sires in 6,384 herds and thus was too large for use in a single analysis. In order to provide an unbiased analysis, six random samples were selected from the full data set by randomly sampling 1,000 herds [14,20]; each sampled herd had to contain at least 1,000 test day records. Each sample contained approximately 15,000 to 20,000 sires and 400,000 to 450,000 cows. These six sub-samples were used for the estimation of the variance components in the model discussed below.

A selected data set was created and consisted of data concerning only the specific 383 sires of interest and their offspring. This data set contained 333,068 Holstein Friesian daughters with 2,311,834 records and was used to estimate the sire effect EBV for persistency and total milk yield (incorporating information based on the six sub-samples). A maternal-grandsire pedigree dating back to 1940 and consisting of 2864 animals was available for the 383 sires.

A total of 9918 SNP markers were scored on the 383 sires using Parallele (Affymetrix, Santa Clara, CA). After adjusting for monomorphic SNP, missing genotypes, unknown location, minimum allele frequency (> 2.5%) and deviation of observed genotype frequencies from expected frequencies calculated from allele frequencies (Hardy Weinberg equilibrium), the number of polymorphic markers amounted to 7541 with an average of 251 SNP per chromosome (29 autosomes plus one sex chromosome). The remaining missing values in the SNP information were replaced by their expected value calculated using haplotypes of five SNP markers [21].

### Statistical methods

NCSS were used to model the sire influence on lactation curves of dairy cows in the randomly sampled data and also in the selected data set. The randomly selected data sets were used to estimate variance components in the model discussed below. The six sets of estimates were averaged and all but one (as discussed later) of the variances components were fixed at their average value in the analysis of the selected data set. The aim was to reduce the bias in using the selected data by ensuring that the variance component estimates reflected those that would be obtained if the full data was analysed.

For the analysis on the selected data, the main features of the lactation curves were extracted. The sire's influence on the peak lactation milk yield and the corresponding day of peak milk yield were estimated, and for each sire, the EBV for persistency and total milk yield were subsequently computed. This constituted the first stage of analysis.

Then, the EBV for persistency and total milk yield were used in the second stage association study. Appropriate weights were calculated for the second stage analyses, reflecting the information available for each sire. A discussion of weights for two-stage analysis has been presented by Smith and colleagues [22] in the context of plant breeding but the methods are more widely applicable and relevant for the analyses conducted in this paper.

#### Stage I model

A mixed model was used for both the sampled and selected test day data, namely(1)

The vector **y **is the *Nx*1 vector of test-day milk yields on the cows in both the randomly sampled and the selected data sets. The fixed effects were given by **X**_0 _**τ**_0_, and consisted of trends for the age of cow at test (a fixed effects cubic polynomial) and a fixed effect for year by season; a factor of 46 levels representing year by season interactions. The random effects in the model included herd-test-day effects represented by **u**_0*h *_(with design matrix **Z**_0*h*_), independent effects with mean zero and variance , and the random cubic orthogonal polynomial regression coefficients for the *c *cows in the data are given by **u**_0*c *_(with design matrix **Z**_0*c*_), with mean zero and variance matrix **G**_0*c *_⊗ **I**_*c*_; **G**_0*c *_is a 4 × 4 variance matrix (⊗ is the Kronecker product). The random cubic regression using orthogonal polynomials was included to model cow lactation across the repeated measures of milk yield over the lactation period and it incorporates permanent environmental effects and genetic effects since the maternal grandsire pedigree was not included in the stage I model. It would have been preferable to include the pedigree in this first stage of modelling, especially if EBV were of prime interest since they would then reflect relationships between sires, but we were unable to do so due to limitations in computing power. However, the pedigree was used in the association analysis discussed and presented below. All random effects were assumed to have a normal distribution and to be mutually independent. The error term was assumed independently distributed as *N*(0, *σ*^2^**I**_*N*_).

The term **Zg **represents the sire effects on lactation over time. Thus **Z **is a design matrix for the sire of cow effect. The vector **g **is the vector of sire contributions to the lactation curves of the cows. Thus **g **can be partitioned into components that correspond to individual sires; that is  for the 383 sires for the selected data set.

The contribution to the lactation curve of cows for the *j *th sire, was modelled using NCSS [2,23], that is (*j *= 1, 2, ..., 383) as(2)

where the spline is represented by a fixed linear (or straight line) component, **X**_*s*1 _**τ**_*js*_, and a correlated random component, **Z**_*s*1_**u**_*js*_, to allow for nonlinear patterns in the lactation curve attributable to sires. Note that **u**_*js *_~*N*(**0**, **I**_*n*-2_) uses the formulation of Verbyla and colleagues [23], where  is the variance component for the random component of the NCSS and *n *is the number of knot-points for the NCSS. The same knot points were used for all sires. The full design matrices for  and  in (1) become respectively, **X**_*s *_= **Z**(**X**_*s*1 _⊗ **I**_383_) and **Z**_*s *_= **Z**(**Z**_*s*1 _⊗ **I**_383_) for the 383 sires in the selected data set.

Notice that the cow random coefficients and NCSS provide for the variance-covariance structure that would arise because of repeated measurements on the individual cows.

The full model is given by(3)

and the marginal distribution of **y **is therefore given by

where **Xτ **= **X**_0 _**τ**_0 _+ **X**_*s *_**τ**_*s *_are the fixed effects, and the variance matrix **H **is given by

It was possible to fit this model, whereas more complex models (for example allowing for splines for each cow) were simply too large to be fitted.

#### Smoothing spline

The key component of the statistical model is the NCSS, one for each sire. This term formed the basis of the analysis of the milk yield characteristics that were influenced by the choice of sire. Once the mixed model (3) is fitted, the sire NCSS can be used to determine the peak milk yield, the time at which the peak occurs, milk yield persistency, and total milk yield over the full lactation.

Some basic results involving NCSS are required in order to determine peak yield, persistency and total milk yield. The first derivative is required to determine the day of peak milk yield. NCSS can then be used to find the peak milk yield value for each sire. The total milk yield is the area under the NCSS for each sire and requires integration of the NCSS.

Suppose we have a quantitative explanatory variable *t *with corresponding values or knot-points *T*_*L *_<*t*_1 _≤ *t*_2 _≤ ... ≤ *t*_*n *_<*T*_*R *_on an interval [*T*_*L*_, *T*_*R*_]. In our context, this variable is DIM, and the interval is [6,305]. Selection of the knot points *t*_*i *_is discussed below.

Suppose that g_*j*_(*t*_*i*_) is the value of the NCSS for the *j*th sire at the knot-point *t*_*i*_, which represents one value of the vector **g**_*j*_. To simplify the notation we drop the subscript *j*. Green and Silverman [24] have shown that the values *g*_*i *_= *g*(*t*_*i*_) and the second derivatives *γ*_*i *_= *g*"(*t*_*i*_) at the knot points *t*_*i *_characterize the NCSS; note that *γ*_1 _= *γ*_*n *_= 0. In fact, for *t*_*i *_≤ *t *≤ *t*_*i*+1 _and *h*_*i *_= *h*_*i*+1 _- *t*_*i*_,(4)

While the terms in (4) do not match the formulation in (2), White and colleagues [25] have shown the equivalence of various forms on the NCSS. Equation (2) is useful in fitting models in statistical software packages, whereas (4) is useful for post-fitting calculations.

Several results are needed to develop the second stage of the analysis, namely the association study. Equation (4) can be written as

where **g **and **γ **are vectors of the *g*_*i *_and *γ*_*i*_, respectively, and **a**_1 _and **a**_2 _are known vectors explicitly defined using (4), and which are equal to zero, apart from the two indices *i *and *i *+ 1. Using equation (2.4) of [24], we can then write(5)

where **Q **and **R **are known matrices given on pages 12 and 13 of [24]; **Q **and **R **are functions of *h*_*i*_. Thus any value of the function *g *can be found using the values at the knot points.

Using (4), the first derivative of *g*(*t*) can be shown to be (*t*_*i *_≤ *t *≤ *t*_*i*+1_)(6)

where

and

Equation (6) is used to determine the time for maximum or peak milk yield.

#### Peak lactation and persistency

Typically, there is a single maximum or peak milk yield day at which . The first step is to use the spline to determine the interval containing the peak milk yield. In most cases, the interval containing peak values has first derivatives at the knot points satisfying  and  where the hat indicates the estimated *g*; if there is no turning point in the lactation curve, the maximum will occur at the initial time point and there will not be any interval satisfying the inequalities. Once the interval containing the maximum milk yield is determined, the equation  is solved and involves finding the acceptable root of the quadratic equation (6).

Estimated persistency was calculated as the difference between the milk yield at peak lactation and an end day, namely(7)

where *t*_max _and *t*_*end *_are the time of peak milk yield and the end time (*t*_*end *_= 305 DIM) respectively. The time period differs between sires because of differing peak lactation times *t*_max_. The estimated milk yields  and  were calculated for each sire using (4).

Both variability of the actual time of peak yield attributable to sires and difference in persistency were examined using a fixed time (60 DIM). Relationships between peak lactation time, peak lactation value, lactation at the end of the lactation period, persistency and total milk yield were also examined.

#### Total milk yield

The total milk yield for cows attributable to sires was found by calculating the area under the NCSS for each sire. The area under the curve can be found by integration,

and using (4) it is easy to show(8)

Evaluation of (8) for each sire involves using estimates of *g*_*i *_and *γ*_*i*_, and using the same arguments leading to (5), can be written in terms of *g *at the knot-points as(9)

where the **b **vectors are functions of *h*_*i *_as given in (8).

#### Weights for stage II analysis

The association analyses are conducted in the second stage of the analysis. However, the 'data' for the second stage are estimates or predictions from stage 1 and hence have an associated error that should be carried through to the next stage of analysis. These estimates are also correlated, but to provide a simple analysis, an approximation along the lines of [22,26] is carried out. The weights are determined as follows.

The predicted persistency involves finding  and . Thus for a single sire, and using (5),

where  is a known vector. The variance matrix of **ĝ**, which we denote by **V**, is available via the prediction error variance matrix, and the underlying spline variance matrix as outlined [23].

If **A**_*c *_is the matrix whose rows are given by , and using the ideas in [22,26], our weights are given by(10)

the diagonal elements of the inverse of the full variance matrix of the persistency estimates. Note that (10) ignores the error associated with estimating *t*_max_.

The same argument was used to develop weights for the total milk yield estimates using (9).

#### Stage II model

We examined additive SNP marker associations for both persistency and total milk yield using the methods of Kiverii [16,17] with a component of the method discussed by Verbyla and colleagues [18]. Including the polygenic effects using the maternal-grandsire pedigree, with the resulting additive relationship matrix, was also shown to be important.

The statistical model for marker-trait association was given by(11)

where **y**_*m *_is the vector of estimated effects for a single trait (m stands for persistency or total milk yield) from the first stage of the analysis, **1 **is a vector of 'ones', *μ *is an overall mean effect, **M**_*a *_is a matrix of additive SNP scores (see below) with associated size vector **β**_*a*_, **a **is a vector of (polygenic) additive random effects with distribution , where **A **is derived from the full maternal grandsire pedigree and **e**_*m *_is a residual vector distributed as  where **W**_*m *_is a diagonal matrix of weights derived from the first stage of the analysis using (10). Note that **W**_*m *_is a known matrix for this second stage of the analysis and is different for each of the two traits, persistency and total milk yield.

The additive (*m*_*a*_) scores for a SNP with alleles *A *and *B *are given by -1 for genotype *AA*, 0 for genotype *AB *and 1 for genotype *BB*. Thus **M**_*a *_contains the scores *m*_*a *_for each SNP for each sire.

The GeneRaVE or genetic random variable elimination approach presented by Kiiveri [16,17] was used for the analysis without the polygenic effects **a**. The current theory and implementation of GeneRaVE does not allow random effects to be included. Ideally the polygenic effects should be included. Indeed ignoring them would produce a biased selection since it is likely that truly non-significant markers would be selected because the between sire stratum of variation is omitted. However, in order to at least partially correct for the bias, a further stage of analysis is described below. Thus for selection of SNP markers, (11) became

If *β*_*j *_is the size of the effect of the *j *th SNP, the model developed in [21,22] was

so that the size effects conditional on a variance parameter (*v*_*j*_) follow a normal distribution and hence are random effects. The variances were assumed to follow a gamma distribution with shape parameter *k *and scale parameter *b*. This formulation leads to a complex marginal distribution for *β*_*j *_which is a function of |*β*_*j*_|. The dependence on the modulus leads to sparse regression variable selection by enabling estimates of size to be exactly zero. In practice, this was accomplished by setting *β*_*j *_equal to zero if the absolute magnitude was below 10^-6^.

To control for false positives, a 10-fold cross-validation approach was used to find optimal values for the parameters *k *and *b*. An additional scale parameter can also be optimised in the cross-validation. This parameter scales the response so that the threshold of 10^-6 ^is relative to a common scale over different traits. The cross-validation involved sub-dividing the data into 10 random groups, leaving out each group in turn, and predicting the response for that group using the SNP selection process with the nine remaining groups as the data set. The minimum mean square error of prediction across all cross-validations was used as the criterion for selecting *k*, *b *and the scale (denoted *b*0*sc *in the GeneRaVE documentation and in the results section).

In 2007, Verbyla and colleagues [18] presented a method for QTL analysis using a forward selection approach with a simpler random effects model for the sizes. The variances *v*_*j *_were assumed to be equal and non-random. In their approach, QTL were moved to the fixed effects part of the model since they were determined. In this paper, we used Kiiveri's [16,17] selection approach in conjunction with the approach reported by Verbyla and colleagues [18], which consists of moving the complete set of selected SNP to the fixed effects part of the model. The non-selected SNP were omitted in subsequent analyses. At this point, we were also able to include the pedigree information. Thus equation (11) was used for the final analysis, but **β**_*a *_was the vector of sizes only for the selected SNP and the matrix **M**_*a *_contained the additive scores only for the selected SNP.

The significance of the selected SNP was conducted using a standard Wald statistic, namely the estimated SNP size effect divided by the corresponding standard error. Approximate p-values were determined using a standard normal distribution. The resulting significant SNP were used with NCBI *Bos taurus *build Btau_4.0 to construct a list of possible candidate genes [27].

### Computation

The statistical model given by (3) was fitted using ASREML [28] and included lactation curves attributable to the sires in the sub-sampled and selected (383 sires) data sets. The spline term **Z**_*s*_**u**_*s *_in (3) is automatically constructed by ASREML using the approach outlined in [23]. In ASREML, the knot points used for the NCSS are usually the unique values of the explanatory variable and in this case it would have been each observed DIM. Typically such a dense set of knot points is not necessary. By reducing the number of knot-points, computation and time requirements were kept reasonable. The number and their placement are often empirical, although White and colleagues [2] have suggested that eight knot points is usually sufficient for modelling lactation curves. Druet and colleagues [3] have used six knot points successfully. The knot points were positioned at a subset of 6, 36, 66, 96, 126, 156, 186, 231, 261 and 305 DIM. These knot points were selected empirically on the basis of the expected shape of the lactation curve. The number of knot points examined was 6, 8 and 10. Parameter estimates and predictions based on the model were used for comparison, and it was found that six knot points were sufficient for an accurate representation of the lactation curve. Interestingly, log-likelihoods varied across the number of knot points used, but the stability of parameter estimates was clear for six and eight knot points. The final knot points selected were 6, 36, 96, 156, 231, and 305 DIM.

Estimates of persistency and total milk yield were based on the lactation curves obtained using ASREML and were programmed for calculation in R [29]. This included determination of the interval containing the turning point using (6), the calculation of the day at which peak lactation occurred, also using (6), and the peak milk yield using (4). This enabled the sire component of persistency using (7) to be estimated. The area under the lactation curve as given by (8) was also calculated in the R language. The R code includes the calculation of necessary weights for stage two of the analysis, namely the determination of marker-trait association. The R code is available from the authors.

GeneRaVE is available as the R package RChip from Mathematical and Information Sciences at CSIRO http://www.bioinformatics.csiro.au/survival.shtml and this package was used for selection of markers. The subsequent fitting of selected markers as fixed effects using (11) was carried out using ASREML [28].

## Results and Discussion

### Stage 1 Analysis

The six random samples were used to estimate the variance components for the selected data set analysis. The results of these six analyses were very similar, the differences reflecting the sampling variation. The mean of the variance component over the six random samples for the herd test day was  = 7.00, while the residual variance had a mean of  = 4.115. To determine the cubic orthogonal polynomial random regressions covariance matrix for cows over DIM, the estimated matrices obtained from the analyses of the six random samples were averaged and this average is given in Table 1 (with estimated correlations between the components of the random regression given above the diagonal). These values (,  and the values in Table 1) were fixed in the analysis of the selected data set using only the daughters of the 383 sires and the same mixed model. However, the variance component for the spline term **Z**_*s*_**u**_*s *_in (3) was estimated using the selected data since the focus was on the variation among the 383 sires. The estimated variance component for the spline component was  = 2.93.

**Table 1:** The estimated variances, covariances and correlations for the cubic random regression due to cows used in the analysis of the selected data

	P_0_	P_1_	P_2_	P_3_
**P**_0_	6.48	-0.20	-0.14	0.13

**P**_1_	-1.24	6.24	-0.17	-0.37

**P**_2_	-0.83	-0.97	5.34	-0.06

**P**_3_	0.58	-1.68	-0.26	3.26

The values were found by averaging the results from analyses of six random subsets of the full data set; orthogonal polynomials were used (and are denoted by **P**_0 _to **P**_3_); the diagonal values are the estimated variances, the values below the diagonal are estimated covariances, and the values above the diagonal are the estimated correlations between orthogonal polynomial components

### Spline results: persistency and milk yield

**Figure 1:**
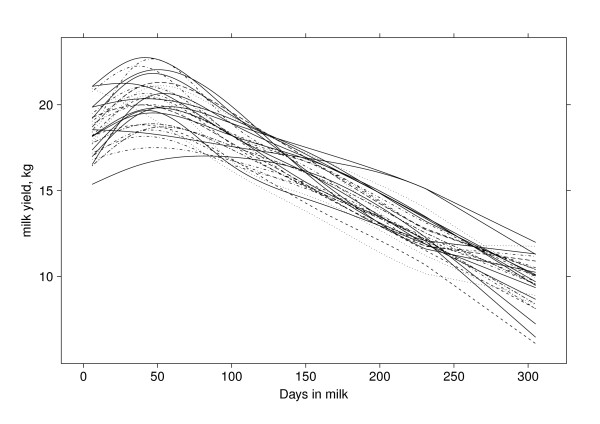
**Sire solutions for the lactation curve found by using the natural cubic smoothing splines**.

In the analysis of the selected data, we found that the estimated milk yield rises to a peak for 369 of the 383 sires and then gradually declines. For the remaining 14 sires, peak yield was estimated to occur at the initial time of 6 DIM. The fitted NCSS for the impact of sire on milk yield are presented in Figure 1 for a (random) subset of 30 sires. The variation in milk yield that is attributable to sires is well illustrated in Figure 1. The estimated lactation curves in Figure 1 all display a decline in milk production post-peak. The post-peak declines vary, and hence display a varying level of persistence. Using a mathematical model for such a diversity of curves could prove to be very restrictive and may miss features found using NCSS.

**Figure 2:**
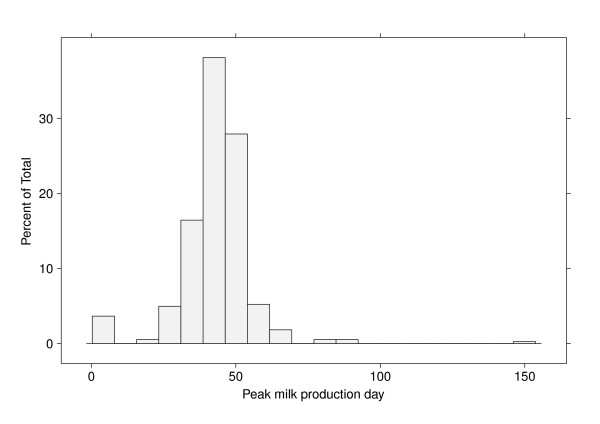
**Histogram of the DIM at peak yield obtained from the sire solutions**.

Potentially, a key aspect of persistency is the timing of peak milk yield. A histogram of the time of peak yield is given in Figure 2 and illustrates the considerable variation (from about 15 to 70 DIM) across sires with a mean time of approximately 40 DIM, rather than 60 DIM which is often used to estimate persistency. Note the single sire outlier at 150 DIM for peak yield. This sire produced an extremely flat lactation curve and was highly persistent after the peak. Persistency was also calculated using the fixed time of 60 DIM for comparison purposes.

**Figure 3:**
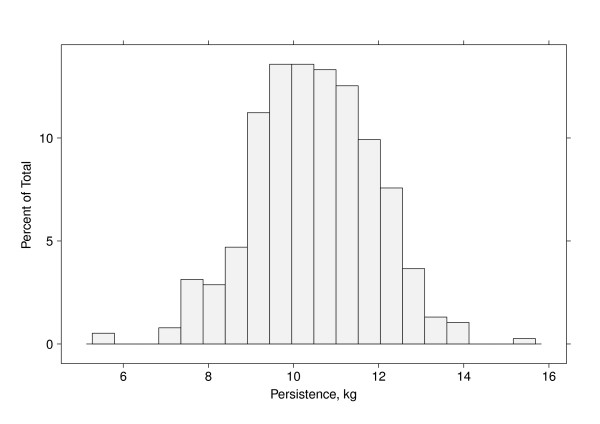
**Histogram of the sire contribution to persistency of milk yield**.

The estimated persistency values (using the actual peak) for the sire effects are presented as a histogram in Figure 3. The distribution showed some skewness to the right indicating that several sires exhibit good persistency (low values), while some sires lead to larger persistency values.

**Figure 4:**
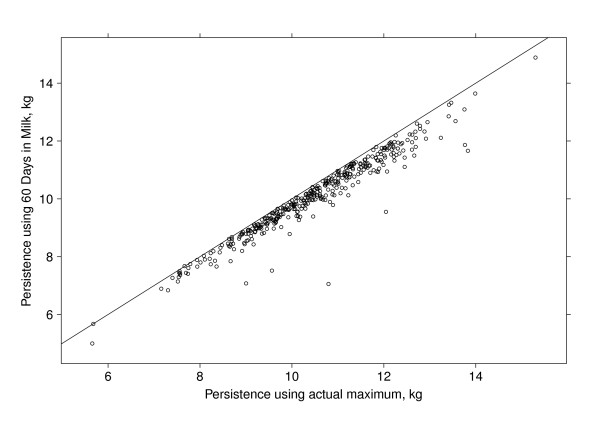
**A comparison of persistency measures**. The figure shows the relationship between the measures of persistency calculated using the estimated actual peak yield for each individual animal and using the fixed 60 DIM yield as peak yield for all animals.

The estimated persistency values based on the estimated peak yield were plotted against the corresponding persistency using the 60 DIM milk yields as the maximum in Figure 4. There was a very strong correlation (0.97) between the two measures. Despite the strong correlation, Figure 4 shows some scatter and re-ranking of values. Notice also that using 60 DIM resulted in a downward bias in terms of estimated persistency (almost all values were below the *y *= *x *line presented). Hence, while the choice of peak DIM may not be totally critical, we favour using the estimated peak whenever possible. However, due to the high correlation between the two measures, the use of the 60 DIM peak yield would seem sufficient in cases where the extra complexity and computational demands cannot be justified.

The definition of persistency used in this paper is one of many possible definitions. Because the peak in milk yield varies across sires, the total time period that defines persistency varies. To examine the impact of the definition of persistency, two further analyses were conducted. First, a fixed time span of 200 days post-peak was used to define persistency. The raw sample correlation between this fixed span persistency and our original measure of persistency was 0.88 while it was 0.90 with the fixed 60 DIM. In the second analysis the original persistency was divided by the time span. The correlation in this case was 0.91 using the estimated peak and 0.99 using 60 DIM. These results suggest a level of consistency across the various definitions of persistency.

**Figure 5:**
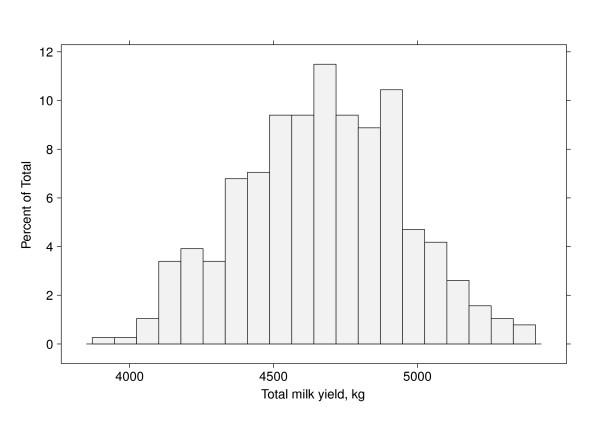
**Histogram of the sire contribution to estimated total milk yield**.

The estimated areas or total milk yields are presented in a histogram in Figure 5. The distribution may be a mixture of a number of components. There may be a genetic reason for this pattern due to the pedigree or SNP markers.

### Correlations

**Figure 6:**
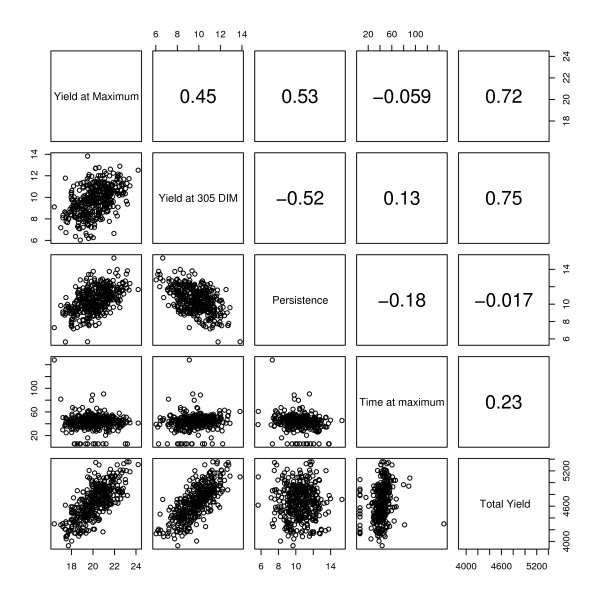
**Scatterplot matrix showing the comparison of the major feature of the lactation curve**. Relationships between peak time, peak yield, yield at 305 d in milk, persistency and total milk yield based on the natural smoothing spline model are plotted and the correlation between these features is also displayed.

The relationships between estimated time to peak, estimated peak value, estimated final value (305 d), estimated persistency and estimated total milk yield (Area) are presented in Figure 6. Total milk yield showed little correlation with persistency. In 2009, Cole and VanRaden [14] reported a similarly small correlation (0.03). It has been shown in previous studies, that the correlation found between total milk yield and persistency is highly variable and dependent on the definition of persistency with both positive and negative correlations, ranging from less than 0 to over 0.50 [14,30].

The DIM of peak yield showed little correlation with any other variable, other than a small positive correlation with total milk yield. DIM of peak yield has been reported as correlated to persistency [8], however in our study, peak yield rather than time of peak yield was highly correlated with persistency (0.53). The definition used here for persistency states that a lower value for persistency indicates a flat lactation curve and a more highly persistent cow. The positive correlation means that the higher the peak the greater the decrease in yield after the peak (low persistency). This result clearly indicates that animals with a lower peak yield are more persistent. This could be explained by a resultant reduction in metabolic stress, in agreement with the findings of Dekkers and colleagues [4]. Figure 1 also shows that a lower peak generally occurs in conjunction with a more gradual decline in predicted milk production, resulting in a more persistent animal. Peak yield was also positively correlated with final milk yield (0.45) and total milk yield (0.72). A high correlation between peak yield and final milk yield has been previously reported [31].

Overall our results support some previous findings, such as peak yield being directly linked to persistency. A higher peak generally means an animal will have a lower persistency. Our findings do not support a correlation between peak DIM and persistency but this may be due to the definition used for persistency here.

### Association study

**Figure 7:**
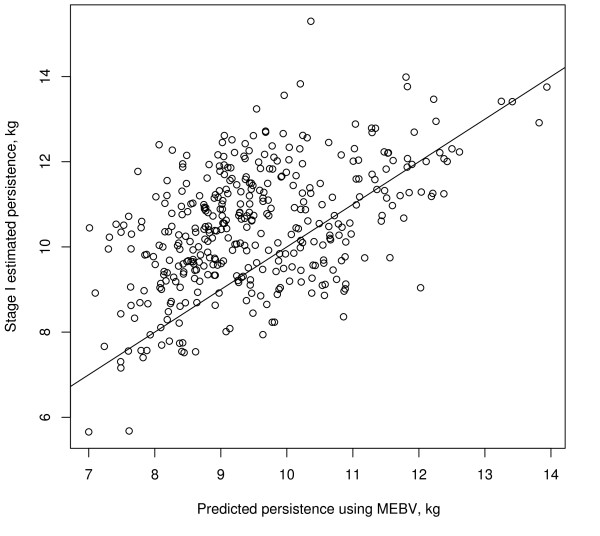
**Comparison of persistency phenotype calculated using NCSS and persistency MEBV using the selected SNP effects and the additive polygenic effect**.

In the GeneRaVE analysis of persistency, the three tuning parameters were set at *b *= 10^7^, *k *= 0 and *b*0*sc *= 0.02 after cross-validation. All three parameters force effects to zero, *b*0*sc *being a scaling factor to help achieve a sparse solution. With these settings (which achieved a low mean squared prediction error) 51 SNP were selected for association with persistency. The selected 51 SNP were moved to the fixed effects part of the model and the remainder of the SNP were discarded. Since a maternal-grandsire pedigree was available for the 383 sires, this was incorporated in the subsequent analysis using (11) with the selected SNP. The estimate of the additive genetic variance was  = 0.76, compared to an average estimated error variance of 0.42; it should be noted that for the association study fixed weights and hence estimated variances from the stage 1 analysis were used at the residual level. Since these vary across sires, an average value is presented to provide an indication of the relative size of additive genetic and residual variation. The pedigree effects have a profound impact on the significance of the selected SNP, because they ensure the appropriate error in testing for significance. The standard errors of the estimated SNP effects when the pedigree was included were two to three times larger than when the pedigree was ignored. Unfortunately, it is not currently possible to include random effects in a GeneRaVE analysis but research is underway to do so. The final 18 SNP that were significant at the 0.10 level are shown in Table 2. Figure 7 is a plot of the persistency EBV calculated using the NCSS against the predicted marker assisted breeding values (MEBV) for persistency. The MEBV was calculated using the significant SNP effects in Table 2 and polygenic effect calculated using the pedigree information. There was a strong correlation (0.95) between the EBV and MEBV but considerable variation still remains unexplained.

**Table 2:** Locations of SNP found significant for persistency

Chromosome	Location (Mbp)	Size	Z ratio	p-value
BTA2	13.2	0.26	2.86	0.0043
BTA2	9.3	0.20	2.29	0.0223
BTA3	95.5	0.22	2.11	0.0351
BTA4	47.8	0.21	1.82	0.0683
BTA4	52.6	0.43	3.27	0.0011
BTA5	8.4	0.34	1.84	0.0659
BTA5	8.2	0.29	2.55	0.0108
BTA6	25.5	0.26	2.29	0.0219
BTA7	84.3	0.35	3.63	0.0003
BTA8	16.6	0.42	3.75	0.0002
BTA10	22.1	0.25	2.54	0.0110
BTA10	62.5	0.30	2.64	0.0082
BTA13	35.5	0.17	1.95	0.0511
BTA14	48.9	0.20	1.69	0.0916
BTA15	51.8	0.31	2.99	0.0028
BTA16	16.3	0.17	1.72	0.0856
BTA28	32.8	0.41	3.62	0.0003
BTAX	70.1	0.22	2.54	0.0112

Selected additive SNP together with the chromosome, the size of the effect on the persistency, the Z ratio (estimate over standard error) and a P-value based on the standard normal distribution; for additive effects, the difference between the homozygotes is twice the stated size value

**Figure 8:**
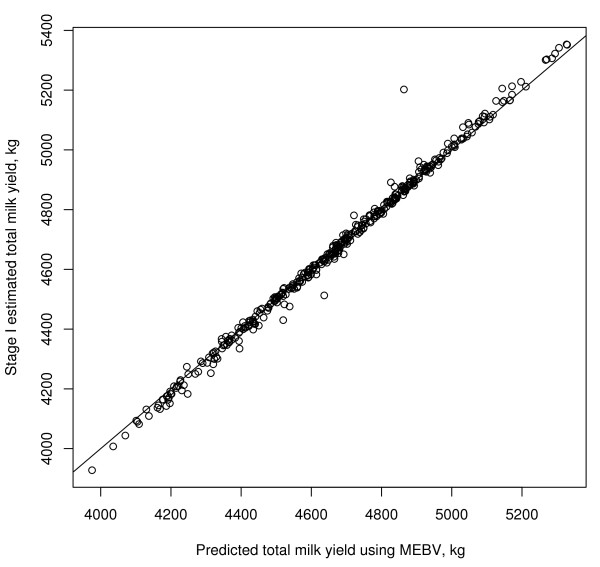
**Comparison of total milk yield phenotype calculated using NCSS and total milk yield MEBV using the selected SNP effects and the additive polygenic effect**.

For total milk yield the GeneRaVE tuning parameters were set at *b *= 10^7^, *k *= 0 and *b*0*sc *= 2.75 after cross-validation. The last parameter reflects the different measurement scale for total milk yield in comparison to persistency. Fifty-two SNP were selected for total milk yield using GeneRaVE. Shifting these putative SNP effects to the fixed effects part of the model and including the pedigree ( = 47, 843 compared to an average estimated error variance of 3,572) reduced the number of SNP to 18 (at the 0.10 level), which are presented in Table 2. Figure 8 is a plot of the observed (using the spline model) and predicted (using the selected SNPs and the pedigree) total milk yields. The correspondence is very good and in fact much better than for persistency (a correlation of 0.996). A single outlier corresponds to a sire with a large weight (from stage 1) and hence lower information content.

In the association mapping study carried out here, we found SNP associations for persistency and milk yield that had previously been reported, as well some newly identified regions or genes that need further analysis.

In the association analysis for persistency, two of the 18 SNP, found significant at the 0.05 significance level (Table 1) are within known genes. There are 14 SNP that appear closely associated with known genes and two other SNP closely associated with hypothetical protein producing loci. One highly significant SNP was found on BTA4 (47.8 Mbp) in the gene CFTR (cystic fibrosis transmembrane conductance receptor) involved in cystic fibrosis in humans. This gene functions as a small conductance chloride channel in epithelial membranes and its function in homeostasis and energy control makes it an ideal candidate gene for involvement in persistency [32].

On BTA15, the SNP appears to associate with the uncoupling protein 3, UCP3, a mitochondrial protein carrier thought to be related to metabolic traits and obesity [33]. Another gene detected in the association analysis of persistency is PAPD1, a polyA polymerase associated domain containing 1. It has been postulated that PAPD1 and UCP3 are involved with obesity and metabolism [34]. Obesity is known to effect lactogenesis [35]. Leptin, a protein hormone produced by adipocytes (fat cells) which has important effects in regulating body weight, metabolism and food intake, has been shown to inhibit hepatocyte growth factor-induced ductal morphogenesis of bovine mammary epithelial cells [36]. It is possible that PAPD1 and UCP3 genes have a similar effect, thereby affecting persistency.

On BTA28, an SNP was significant at the 0.05 level for both persistency and milk yield analyses which suggests an association with the leucine-rich repeat, immunoglobin like and transmembrane domain 1, LRIT1, gene. This region has already been shown to be involved in milk production [37]. There are other significant SNP for persistency that may be associated with known or hypothetical genes and that may be causative, but these need further investigation.

For the total milk yield, the 18 significant SNP are closely associated with known or predicted genes (table 3). The SNP found on BTA1 point to regions already identified as having possible effects on milk yield [38]. This analysis, like the association analysis for persistency, found many SNP in or near genes involved in various functions such as protein binding, signal transduction, receptor binding and membrane stability. The SNP on BTA16 appears to be associated with a gene coding for ATPase, H+ transporting, lysosomal 13 kDa, V1 subunit G3(ATP6V1G3). The SNP on BTA23 and BTA14, respectively, are in regions already shown to have an impact on milk yield [39]. The significant SNP on BTA12, 19 and 24 were in, or close to, genes with known function, but these genes have not previously been associated with milk yield and thus need further investigation.

**Table 3:** Locations of SNP found significant for total milk yield

Chromosome	Location (Mbp)	Size	Z ratio	p-value
BTA1	139.0	84.83	3.67	0.0002
BTA6	22.1	237.30	1.67	0.0940
BTA9	38.1	62.69	1.95	0.0510
BTA11	98.4	162.40	1.75	0.0794
BTA12	34.6	46.71	1.80	0.0714
BTA14	52.8	29.66	1.68	0.0936
BTA19	59.6	77.12	4.36	0.0000
BTA19	14.6	40.22	3.01	0.0026
BTA23	14.8	339.10	2.14	0.0326
BTA23	17.2	82.13	3.59	0.0003
BTA23	13.1	33.12	1.79	0.0728
BTA24	9.1	467.00	2.18	0.0289
BTA24	23.2	74.61	3.57	0.0004
BTA26	23.6	87.41	2.36	0.0184
BTAX	1.5	33.48	1.91	0.0559
BTAX	45.1	289.60	2.06	0.0397
BTAX	71.0	25.64	2.08	0.0374
BTAX	21.1	31.69	1.81	0.0706

Selected additive SNP together with the chromosome, the size of the effect on the total milk yield, the Z ratio (estimate over standard error) and a P-value based on the standard normal distribution; for additive effects, the difference between the homozygotes is twice the stated Size value

## Conclusion

NCSS originally discussed in 1999 by White and colleagues [2] was found very useful to model lactation curves. The methodology described in our paper continues the work of White and colleagues [2] and Druet and colleagues [3] and provides a flexible approach to model lactation curves. The advantage of such a representation is the ease with which important characteristics of the lactation curve such as time to peak, yield at peak, persistency and total milk yield can be determined. Not constraining the curves to have a particular parametric form is also an advantage because it is not necessary that all lactation curves follow the strict form that is implied by such functions.

In our paper, we have extended the use of NCSS for the estimation of EBV of 383 sires for persistency of lactation and total milk yield, two important characteristics of the lactation curve. Sire EBV can be found for both traits allowing the ranking of sires and hence enabling selection and management decisions to be made in practice. NCSS can be used to easily model the sire influence of all the important features of the lactation curve. Importantly, persistency can be calculated using the estimated peak rather than a fixed day across all animals. However, this may not be possible to implement in the situation of a breeding association since the computational demands and the extreme number of records may be too great.

The genome-wide association study found SNP associated with persistence of milk yield and total milk yield that were close to genes of known or postulated function, part of these confirming previous results. The inclusion of the polygenic effect in the analysis was crucial in establishing significant associations. It would be possible to repeat the association study with the genotyped animals using the Illumina Bovine SNP50 chip but it would be necessary to increase the number of genotyped animals to have sufficient power to identify significant QTL.

Lastly, the use of 'sparse' selection tools [16,17] is useful to reduce important SNP to an appropriate number. Despite the successful discovery of SNP related to milk persistence and total milk yield, the association mapping conducted here is largely exploratory and several issues still require further investigation. The first issue concerns additional fixed and random effects that are typically necessary in such an analysis. This is particularly important because pedigree information is often available and the association between genotypes is modelled using an additive relationship matrix through a random effect. Including such information can have a major impact on the association mapping, as shown here when the pedigree was included. The second issue relates to the status of the selected markers. As random effects, they will be shrunk towards zero, while if taken as fixed effects after selection, some bias is likely to occur. The degree of such bias is unknown. These issues are currently investigated by the authors and colleagues.

## Competing interests

The authors declare that they have no competing interests.

## Authors' contributions

Both authors contributed equally to all parts of the study. Both authors have read and approved the final manuscript.
